# Supplementary material for: An Integrated Bioinformatics Analysis Repurposes an Antihelminthic Drug Niclosamide for Treating HMGA2-Overexpressing Human Colorectal Cancer
Source: Cancers (Basel). 2019 Oct 2;11(10):1482. doi: 10.3390/cancers11101482 (PMC6826424; doi:10.3390/cancers11101482)
Supplement: Supplementary file 1 [file cancers-11-01482-s001.zip › cancers-595132-suppl/cancers-595132-suppl.docx]

Supplementary materials:An Integrated Bioinformatics Analysis Repurposes An Antihelminthic Drug Niclosamide for Treating HMGA2-Overexpressing Human Colorectal Cancer

Stephen Wan Leung, Chia-Jung Chou, Tsui-Chin Huang and Pei-Ming Yang


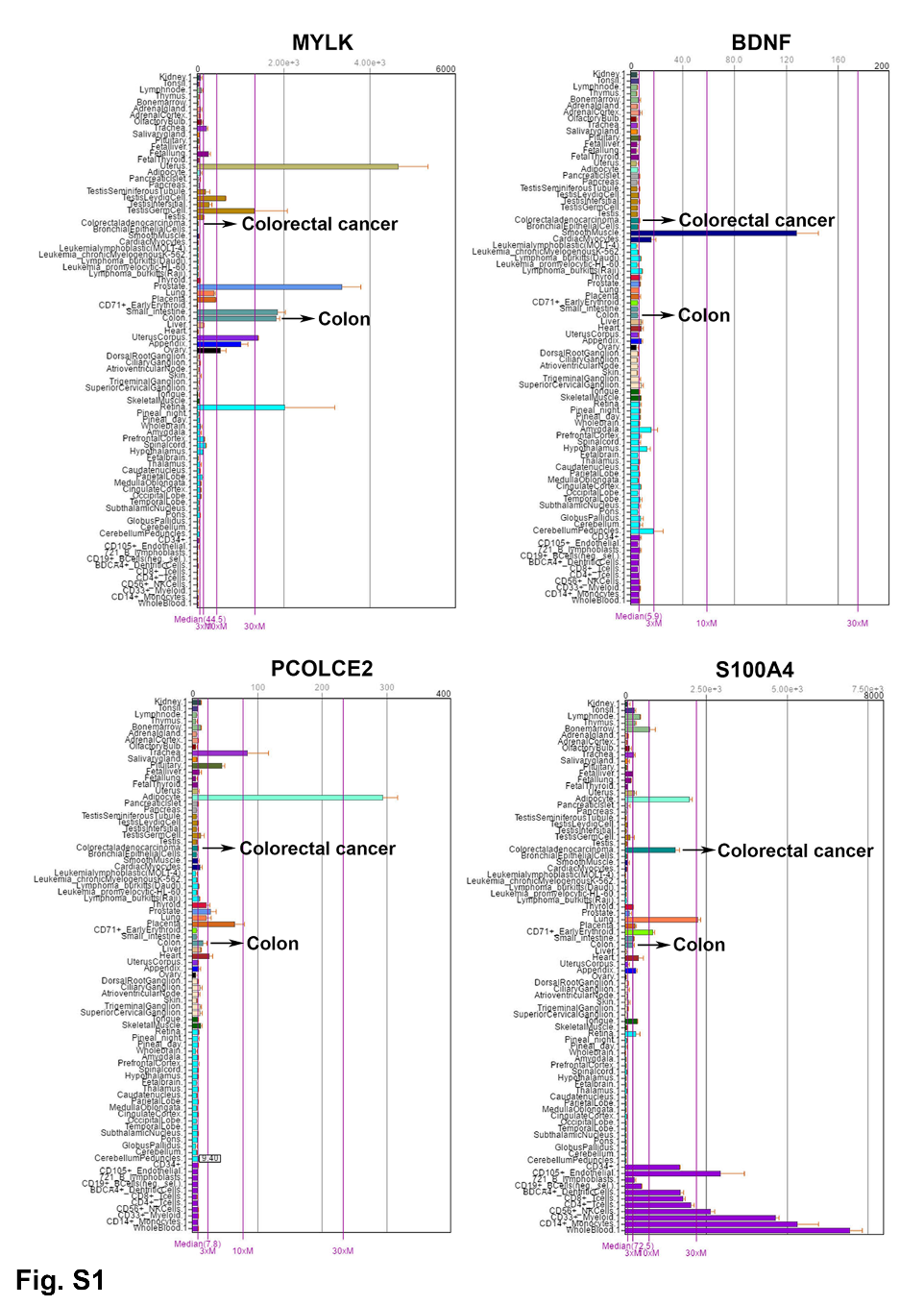


**Figure S1.** Gene expression profiles of *MYLK*, *BDNK*, *PCOLCE2*, and *S100A4* in cell lines.


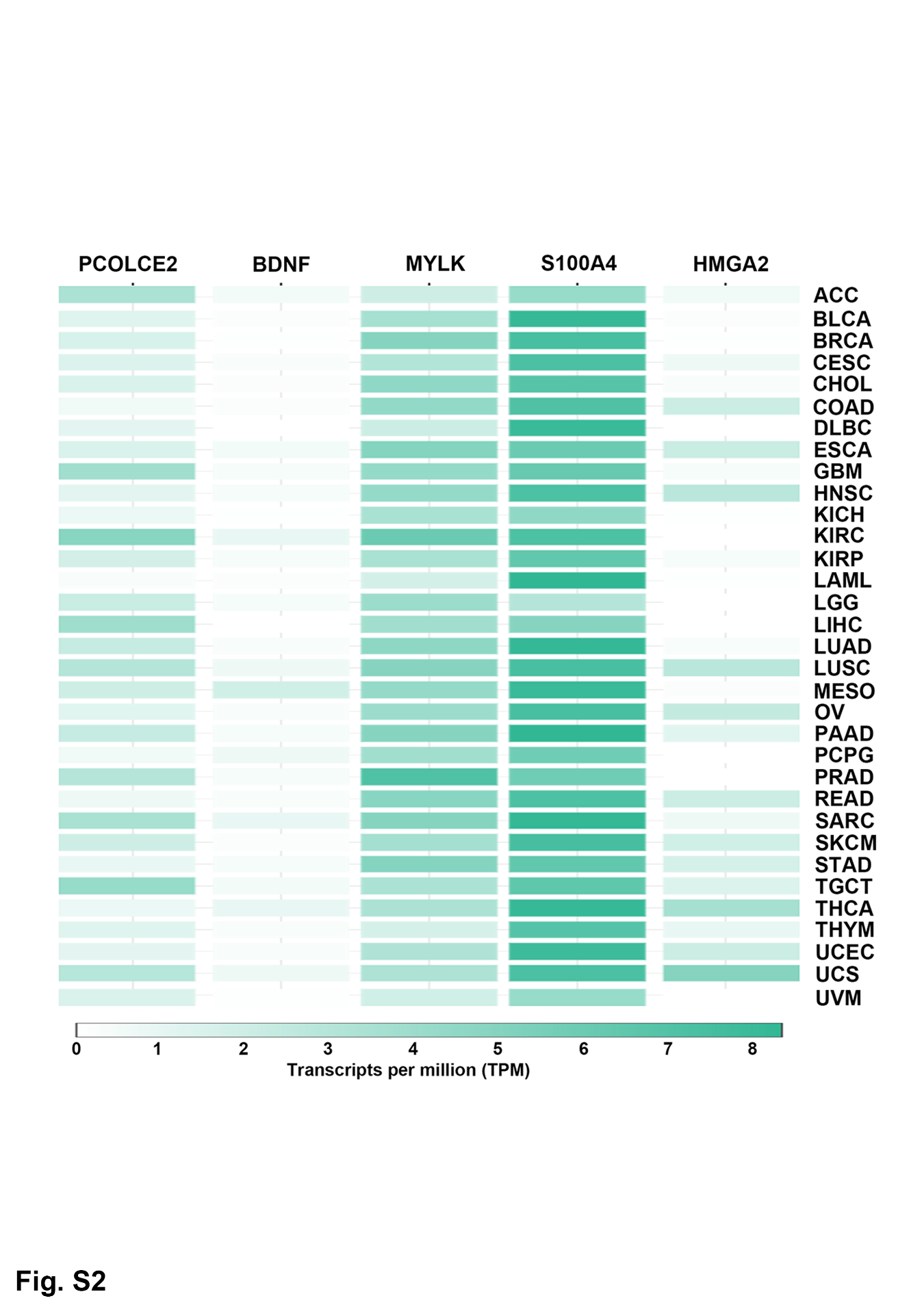


**Figure S2.** Relative gene expression levels of *HMGA2*, *MYLK*, *BDNK*, *PCOLCE2*, and *S100A4* in normal and cancer tissues.


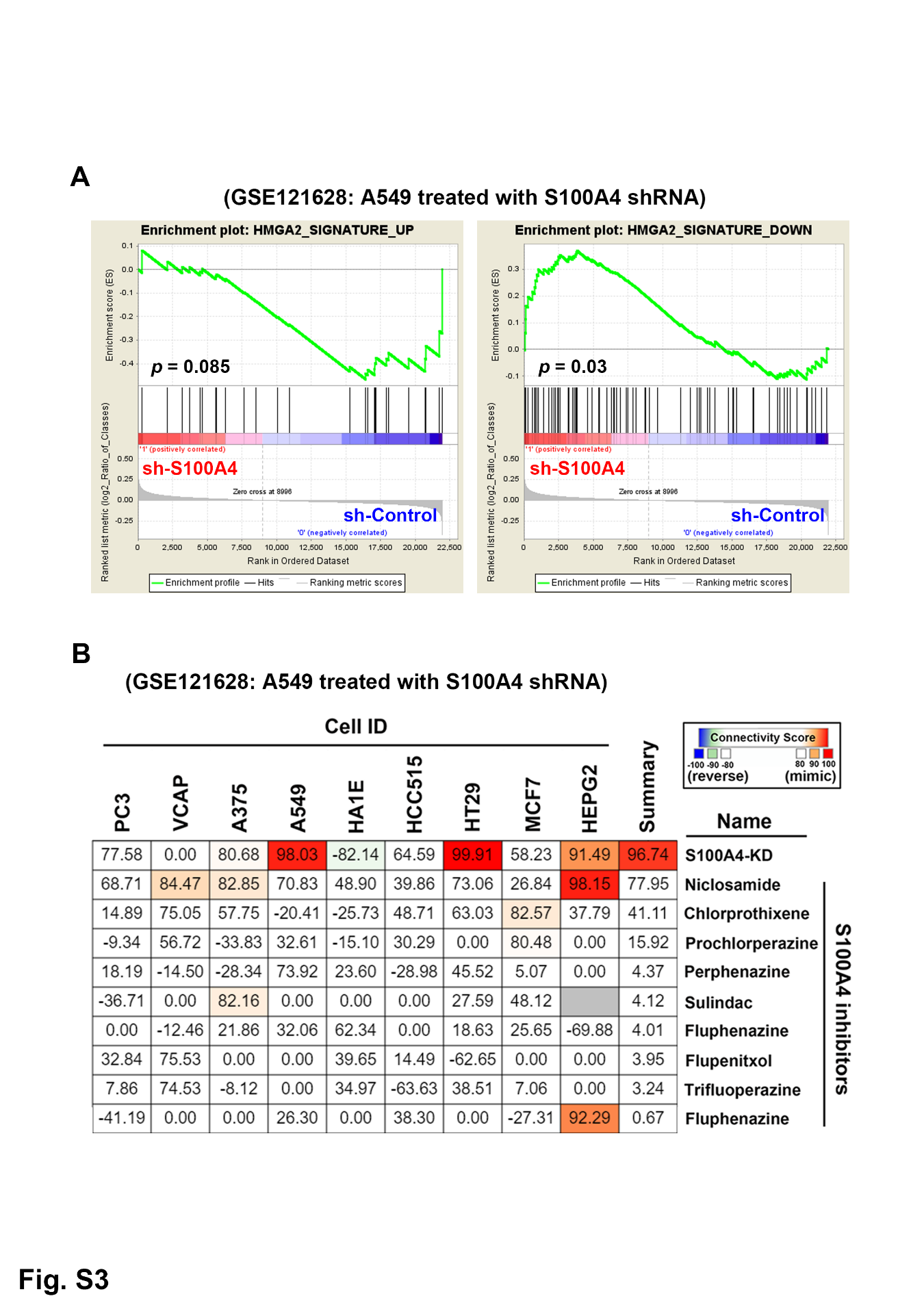


**Figure S3.** Connectivity mapping for the gene signature in *S100A4*-knockdown A549 cells.


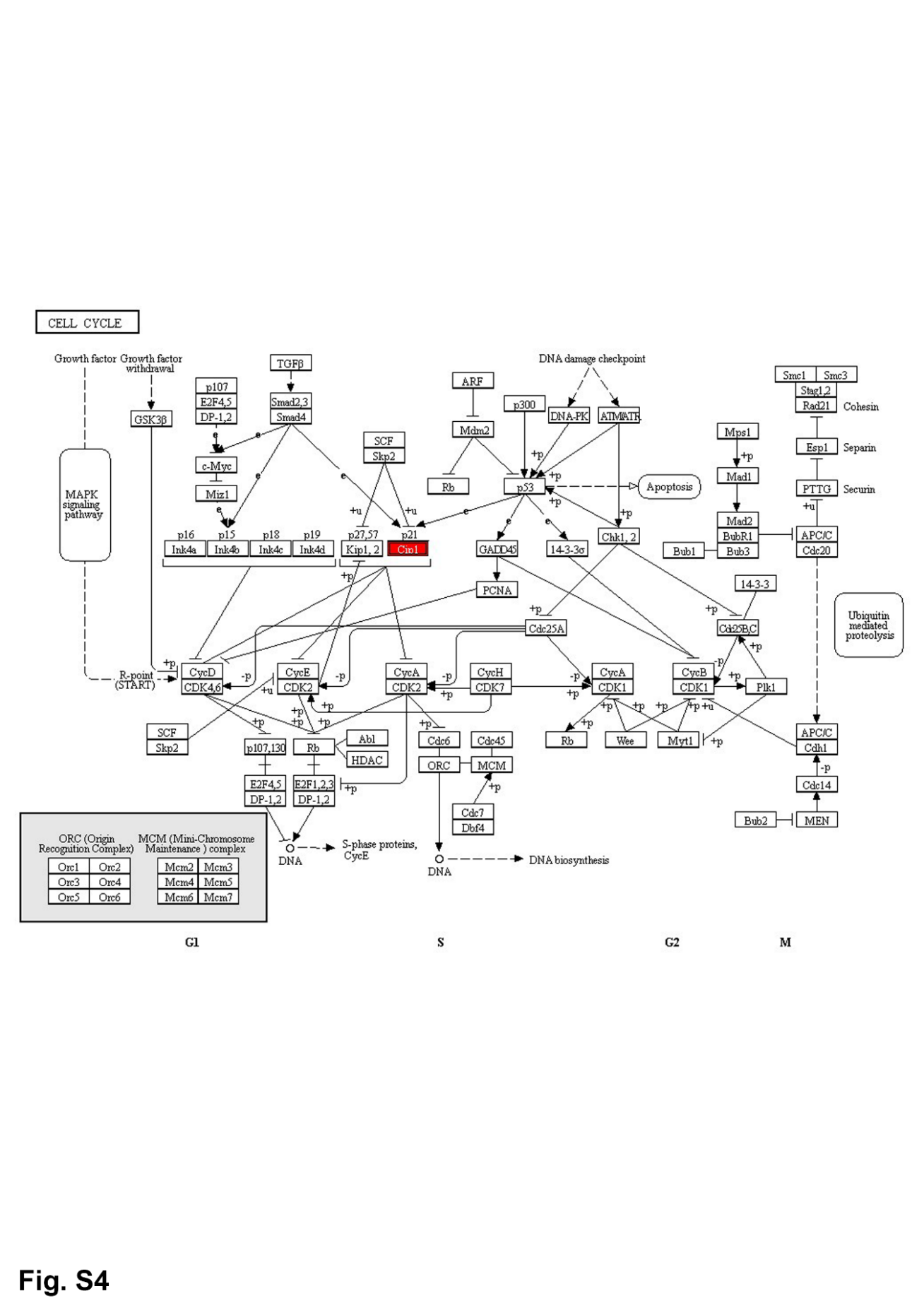


**Figure S4.** The mapping of cell cycle pathway for the upregulated and downregulated genes in niclosamide-treated DLD-1-Vector cells.


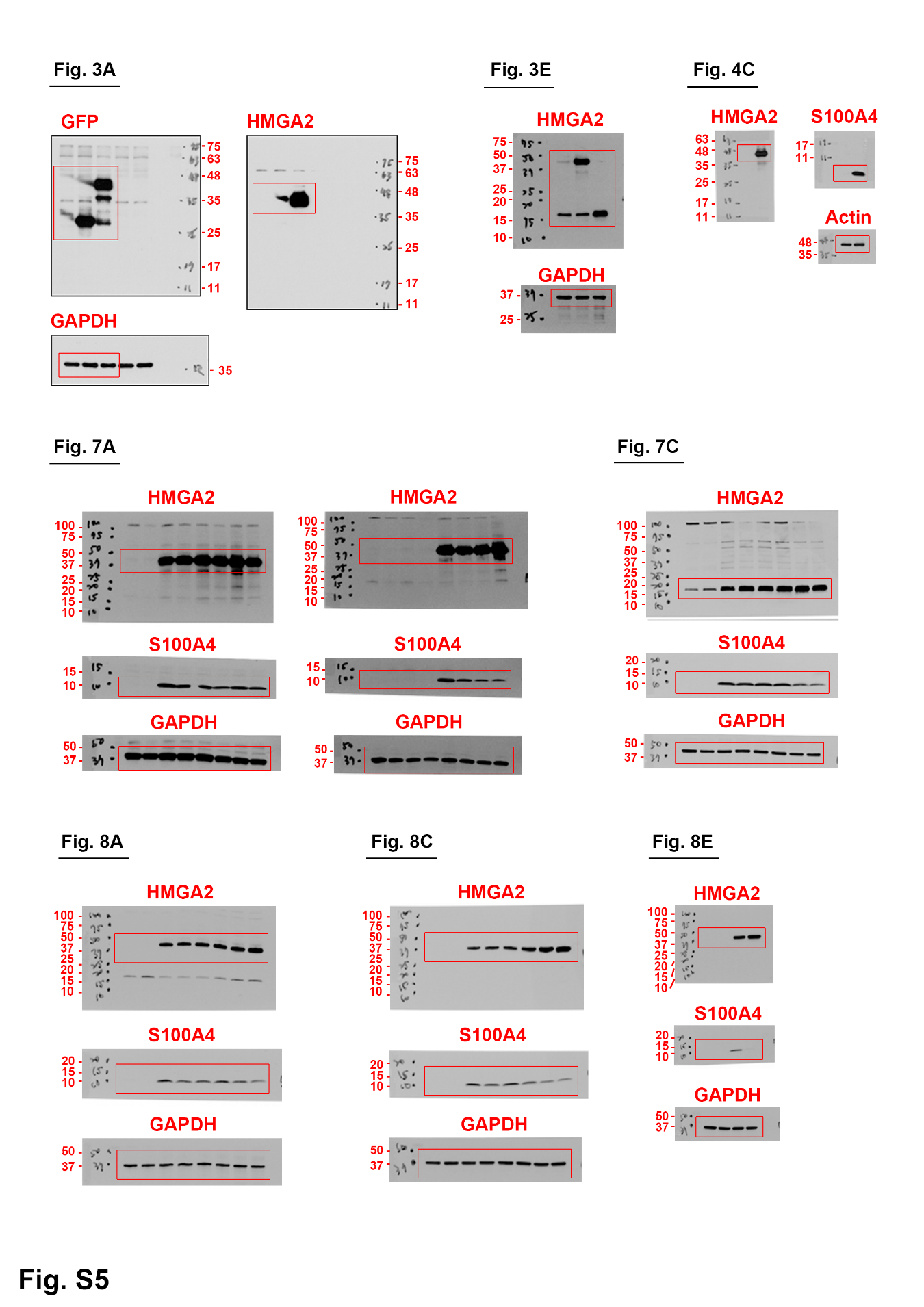


**Figure S5.** The whole uncropped images of the original Western blots.


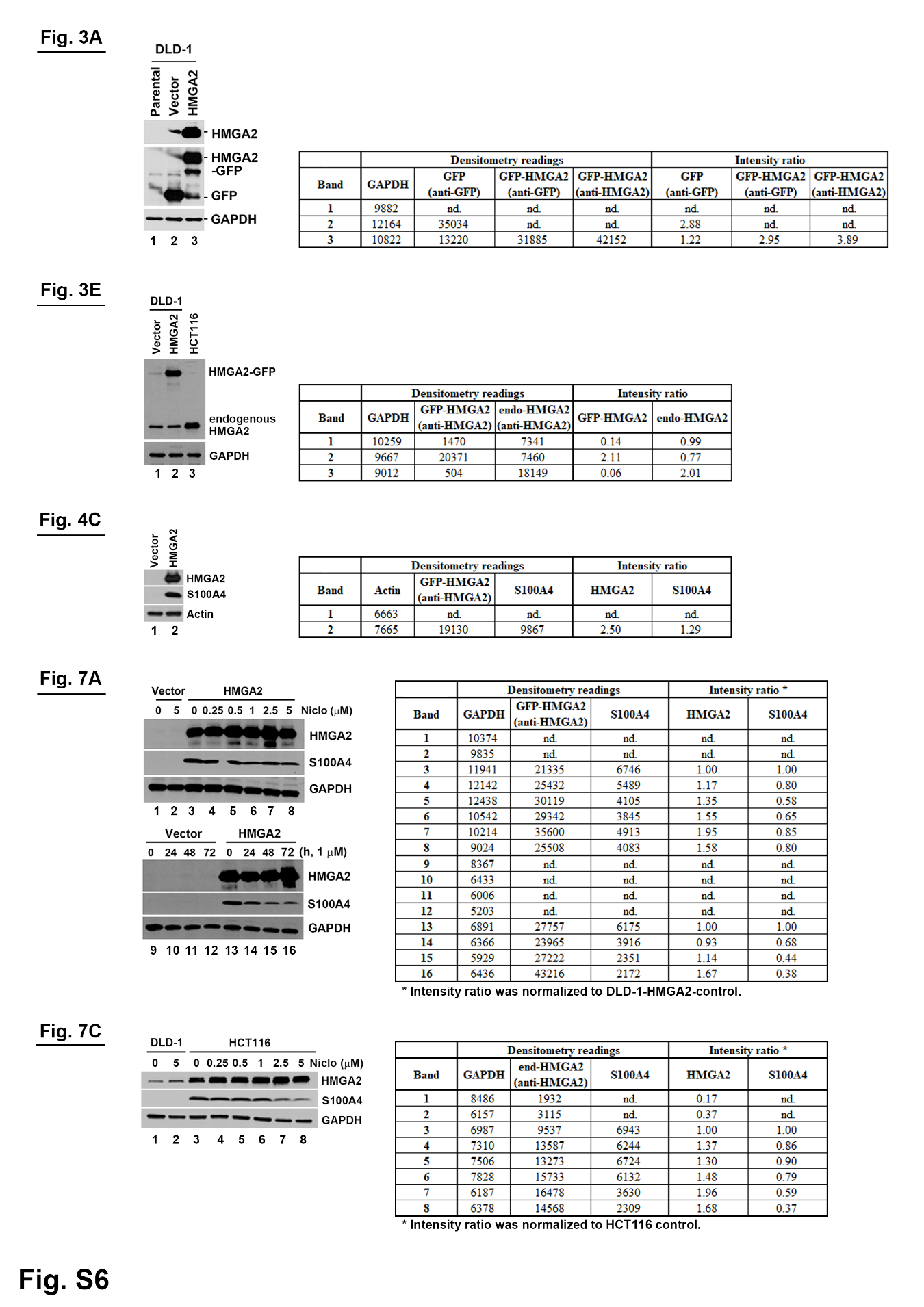


**Figure S6.** The band intensity and ratio for the Western blots in figures.


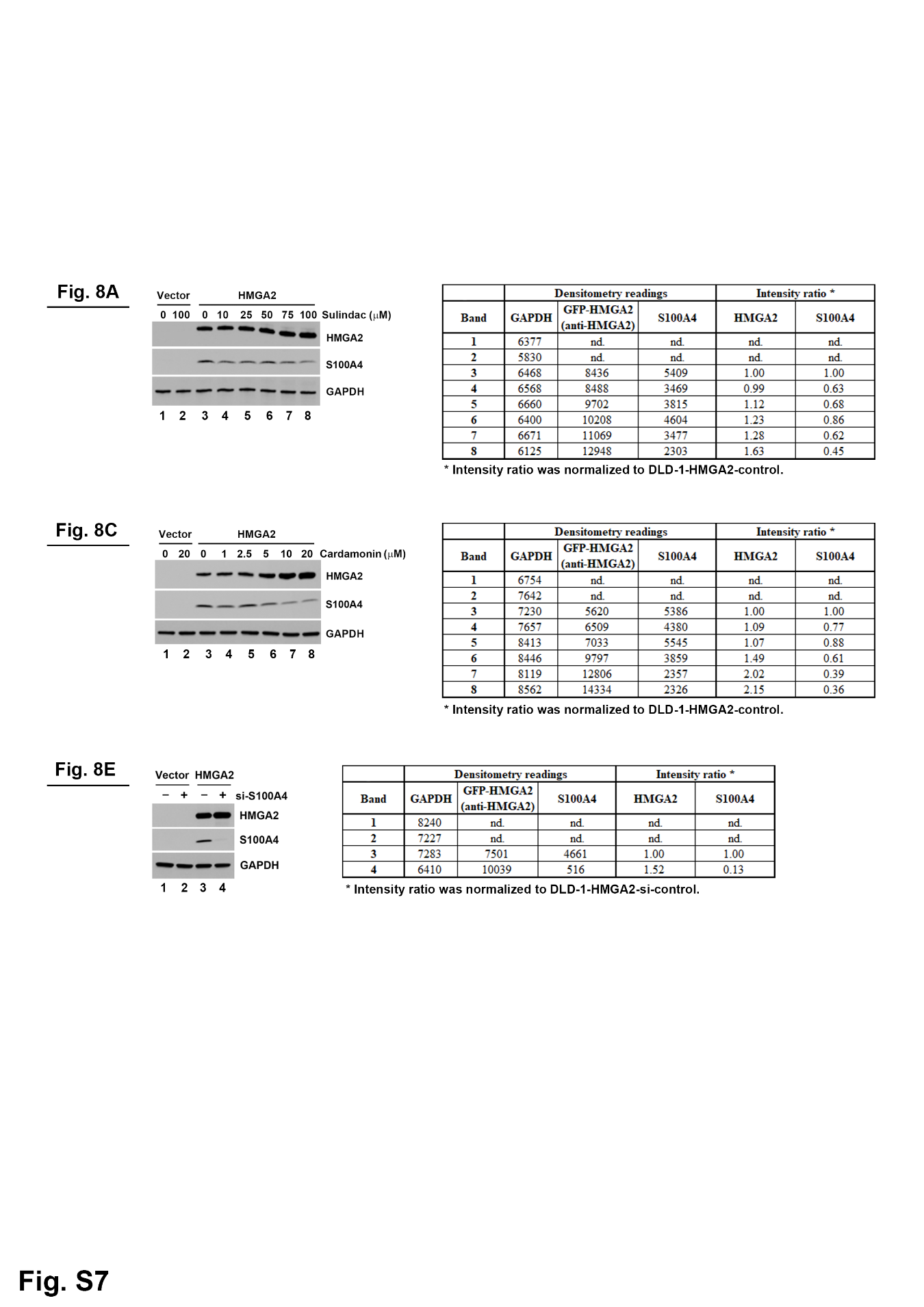


**Figure S7.** The band intensity and ratio for the Western blots in Supplementary Figures.
