# Supplementary material for: An Integrated Bioinformatics Analysis Repurposes an Antihelminthic Drug Niclosamide for Treating HMGA2-Overexpressing Human Colorectal Cancer
Source: Cancers (Basel). 2019 Oct 2;11(10):1482. doi: 10.3390/cancers11101482 (PMC6826424; doi:10.3390/cancers11101482)
Supplement: Supplementary file 1 [file cancers-11-01482-s001.zip › cancers-595132-suppl/cancers-595132-suppl.pdf]

Supplementary materials:An Integrated Bioinformatics Analysis Repurposes An Antihelminthic Drug Niclosamide for Treating HMGA2-Overexpressing Human Colorectal Cancer

Stephen Wan Leung, Chia-Jung Chou, Tsui-Chin Huang and Pei-Ming Yang

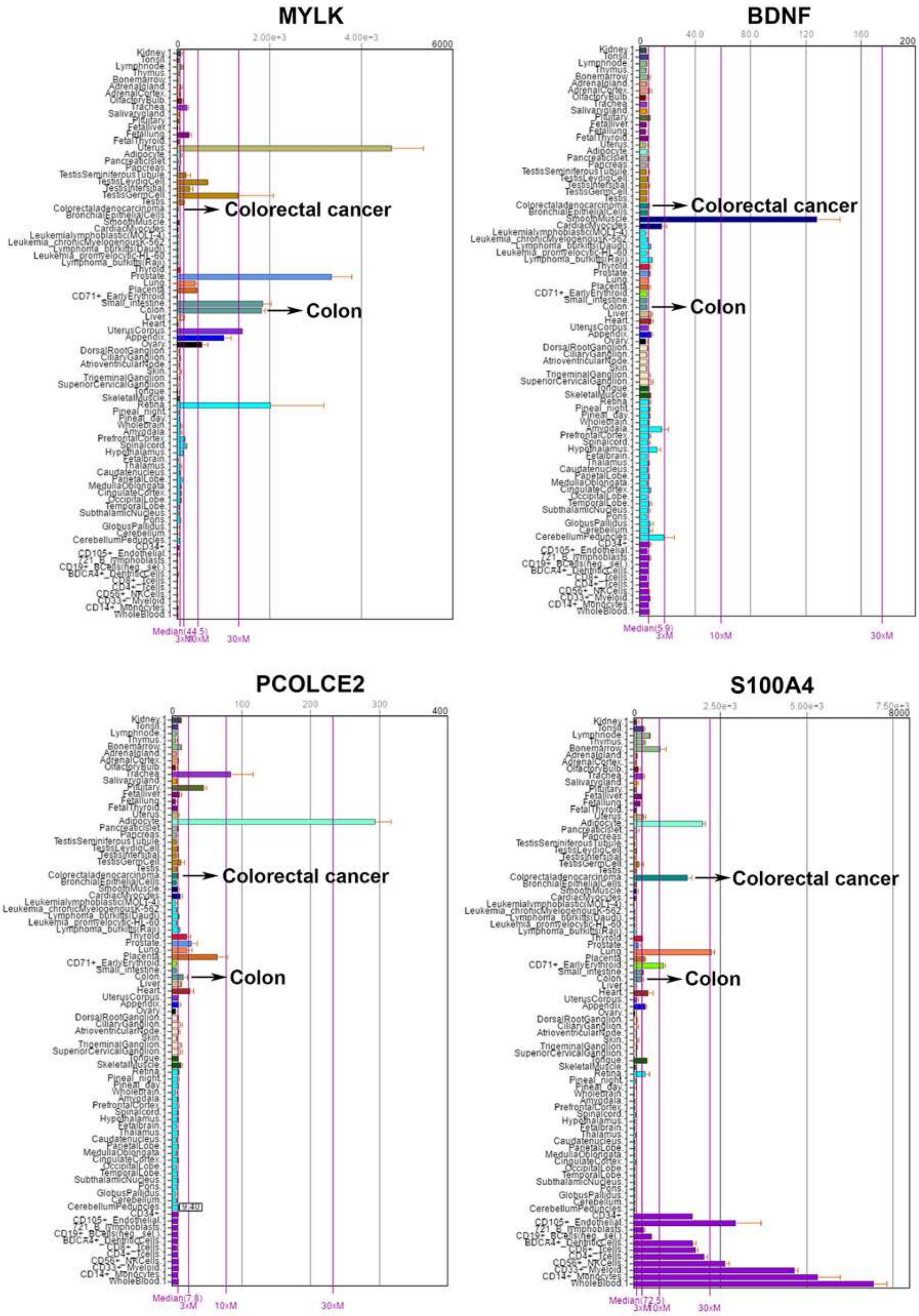

Figure S1. Gene expression profiles of *MYLK*, *BDNK*, *PCOLCE2*, and *S100A4* in cell lines.

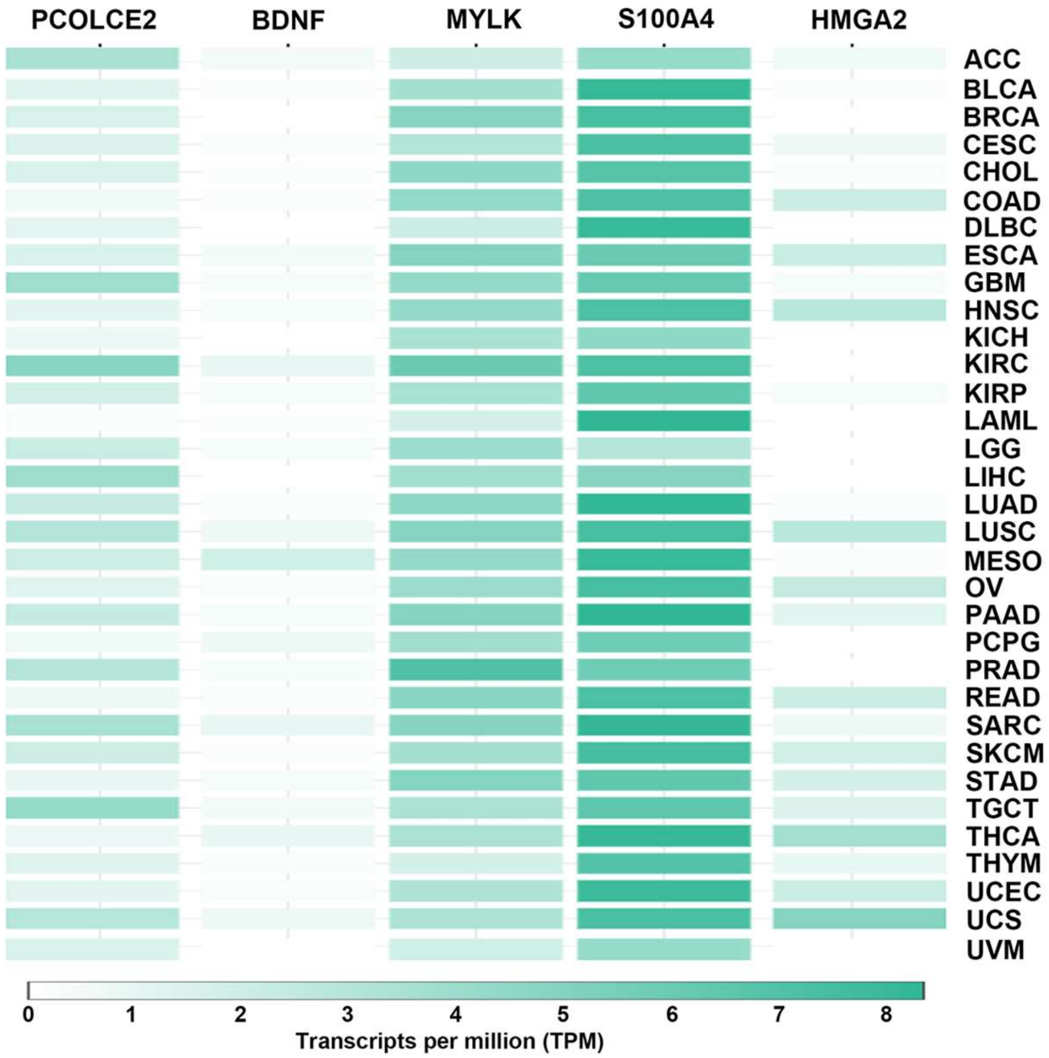

Figure S2. Relative gene expression levels of *HMGA2*, *MYLK*, *BDNK*, *PCOLCE2*, and *S100A4* in normal and cancer tissues.

A

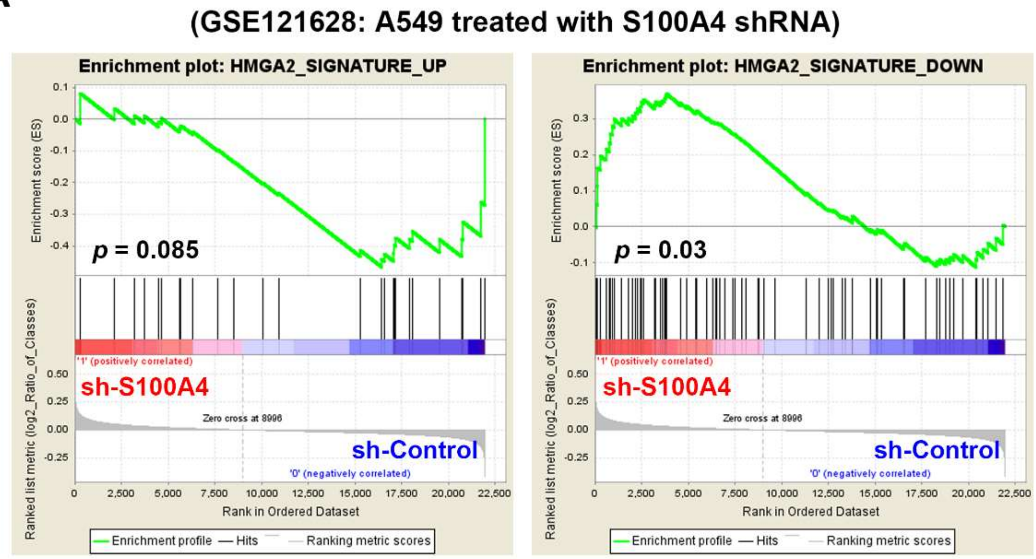

B

(GSE121628: A549 treated with S100A4 shRNA)

Connectivity Score: -100 -90 -80 (reverse); 80 90 100 (mimic)

Cell ID: PC3	VCAP	A375	A549	HA1E	HCC515	HT29	MCF7	HEPG2	Summary	Name	
77.58	0.00	80.68	98.03	-82.14	64.59	99.91	58.23	91.49	96.74	S100A4-KD	
68.71	84.47	82.85	70.83	48.90	39.86	73.06	26.84	98.15	77.95	Niclosamide	S100A4 inhibitors
14.89	75.05	57.75	-20.41	-25.73	48.71	63.03	82.57	37.79	41.11	Chlorprothixene	S100A4 inhibitors
-9.34	56.72	-33.83	32.61	-15.10	30.29	0.00	80.48	0.00	15.92	Prochlorperazine	S100A4 inhibitors
18.19	-14.50	-28.34	73.92	23.60	-28.98	45.52	5.07	0.00	4.37	Perphenazine	S100A4 inhibitors
-36.71	0.00	82.16	0.00	0.00	0.00	27.59	48.12		4.12	Sulindac	S100A4 inhibitors
0.00	-12.46	21.86	32.06	62.34	0.00	18.63	25.65	-69.88	4.01	Fluphenazine	S100A4 inhibitors
32.84	75.53	0.00	0.00	39.65	14.49	-62.65	0.00	0.00	3.95	Flupenitxol	S100A4 inhibitors
7.86	74.53	-8.12	0.00	34.97	-63.63	38.51	7.06	0.00	3.24	Trifluoperazine	S100A4 inhibitors
-41.19	0.00	0.00	26.30	0.00	38.30	0.00	-27.31	92.29	0.67	Fluphenazine	S100A4 inhibitors

Figure S3. Connectivity mapping for the gene signature in *S100A4*-knockdown A549 cells.

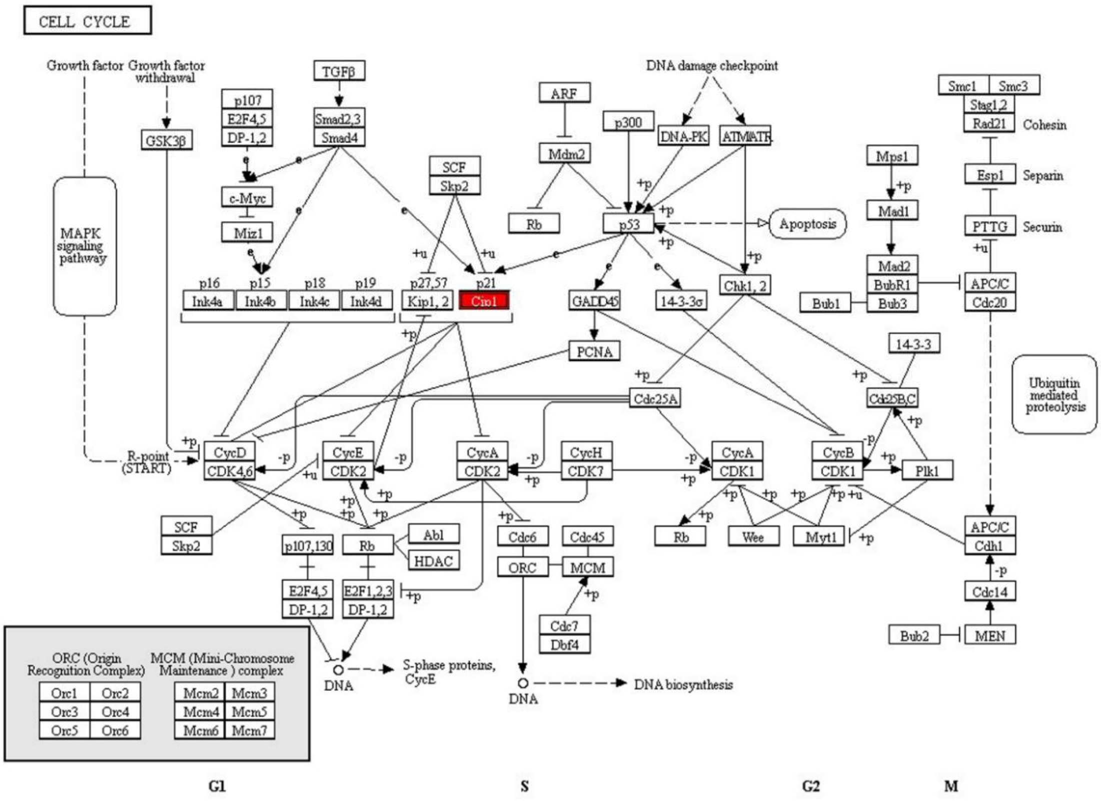

Figure S4. The mapping of cell cycle pathway for the upregulated and downregulated genes in niclosamide-treated DLD-1-Vector cells.

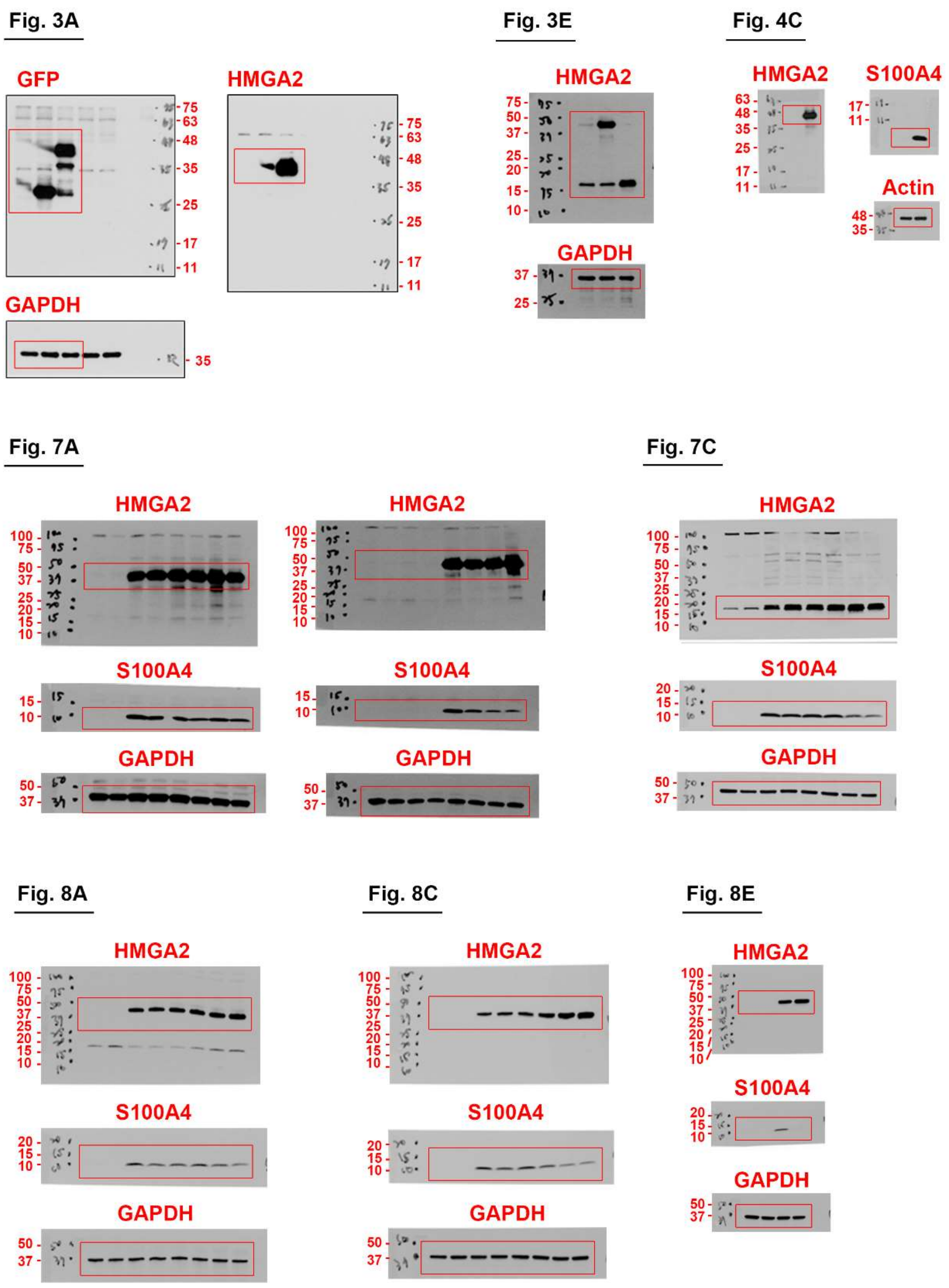

Figure S5. The whole uncropped images of the original Western blots.

Fig. 3A

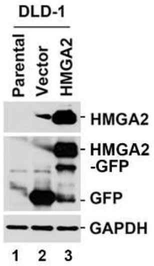

Band	Densitometry readings				Intensity ratio		
	GAPDH	GFP (anti-GFP)	GFP-HMGA2 (anti-GFP)	GFP-HMGA2 (anti-HMGA2)	GFP (anti-GFP)	GFP-HMGA2 (anti-GFP)	GFP-HMGA2 (anti-HMGA2)
1	9882	nd.	nd.	nd.	nd.	nd.	nd.
2	12164	35034	nd.	nd.	2.88	nd.	nd.
3	10822	13220	31885	42152	1.22	2.95	3.89

Fig. 3E

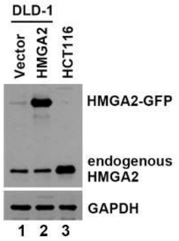

Band	Densitometry readings			Intensity ratio	
	GAPDH	GFP-HMGA2 (anti-HMGA2)	endo-HMGA2 (anti-HMGA2)	GFP-HMGA2	endo-HMGA2
1	10259	1470	7341	0.14	0.99
2	9667	20371	7460	2.11	0.77
3	9012	504	18149	0.06	2.01

Fig. 4C

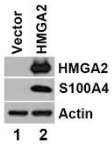

Band	Densitometry readings			Intensity ratio	
	Actin	GFP-HMGA2 (anti-HMGA2)	S100A4	HMGA2	S100A4
1	6663	nd.	nd.	nd.	nd.
2	7665	19130	9867	2.50	1.29

Fig. 7A

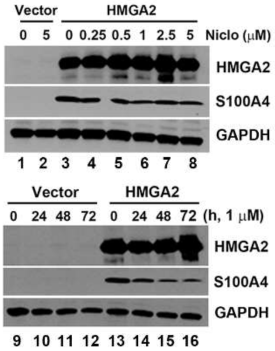

Band	Densitometry readings			Intensity ratio *	
	GAPDH	GFP-HMGA2 (anti-HMGA2)	S100A4	HMGA2	S100A4
1	10374	nd.	nd.	nd.	nd.
2	9835	nd.	nd.	nd.	nd.
3	11941	21335	6746	1.00	1.00
4	12142	25432	5489	1.17	0.80
5	12438	30119	4105	1.35	0.58
6	10542	29342	3845	1.55	0.65
7	10214	35600	4913	1.95	0.85
8	9024	25508	4083	1.58	0.80
9	8367	nd.	nd.	nd.	nd.
10	6433	nd.	nd.	nd.	nd.
11	6006	nd.	nd.	nd.	nd.
12	5203	nd.	nd.	nd.	nd.
13	6891	27757	6175	1.00	1.00
14	6366	23965	3916	0.93	0.68
15	5929	27222	2351	1.14	0.44
16	6436	43216	2172	1.67	0.38

* Intensity ratio was normalized to DLD-1-HMGA2-control.

Fig. 7C

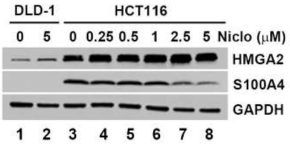

Band	Densitometry readings			Intensity ratio *	
	GAPDH	end-HMGA2 (anti-HMGA2)	S100A4	HMGA2	S100A4
1	8486	1932	nd.	0.17	nd.
2	6157	3115	nd.	0.37	nd.
3	6987	9537	6943	1.00	1.00
4	7310	13587	6244	1.37	0.86
5	7506	13273	6724	1.30	0.90
6	7828	15733	6132	1.48	0.79
7	6187	16478	3630	1.96	0.59
8	6378	14568	2309	1.68	0.37

* Intensity ratio was normalized to HCT116 control.

Figure S6. The band intensity and ratio for the Western blots in figures.

Fig. 8A

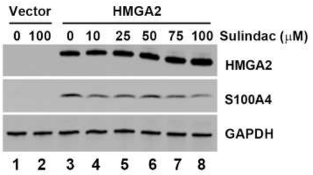

Band	Densitometry readings			Intensity ratio *	
	GAPDH	GFP-HMGA2 (anti-HMGA2)	S100A4	HMGA2	S100A4
1	6377	nd.	nd.	nd.	nd.
2	5830	nd.	nd.	nd.	nd.
3	6468	8436	5409	1.00	1.00
4	6568	8488	3469	0.99	0.63
5	6660	9702	3815	1.12	0.68
6	6400	10208	4604	1.23	0.86
7	6671	11069	3477	1.28	0.62
8	6125	12948	2303	1.63	0.45

* Intensity ratio was normalized to DLD-1-HMGA2-control.

Fig. 8C

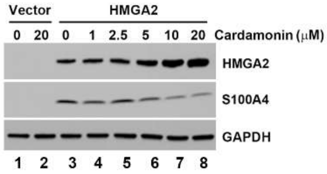

Band	Densitometry readings			Intensity ratio *	
	GAPDH	GFP-HMGA2 (anti-HMGA2)	S100A4	HMGA2	S100A4
1	6754	nd.	nd.	nd.	nd.
2	7642	nd.	nd.	nd.	nd.
3	7230	5620	5386	1.00	1.00
4	7657	6509	4380	1.09	0.77
5	8413	7033	5545	1.07	0.88
6	8446	9797	3859	1.49	0.61
7	8119	12806	2357	2.02	0.39
8	8562	14334	2326	2.15	0.36

* Intensity ratio was normalized to DLD-1-HMGA2-control.

Fig. 8E

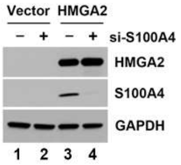

Band	Densitometry readings			Intensity ratio *	
	GAPDH	GFP-HMGA2 (anti-HMGA2)	S100A4	HMGA2	S100A4
1	8240	nd.	nd.	nd.	nd.
2	7227	nd.	nd.	nd.	nd.
3	7283	7501	4661	1.00	1.00
4	6410	10039	516	1.52	0.13

* Intensity ratio was normalized to DLD-1-HMGA2-si-control.

Figure S7. The band intensity and ratio for the Western blots in Supplementary Figures.
