# Supplementary material for: An Integrated Bioinformatics Analysis Repurposes an Antihelminthic Drug Niclosamide for Treating HMGA2-Overexpressing Human Colorectal Cancer
Source: Cancers (Basel). 2019 Oct 2;11(10):1482. doi: 10.3390/cancers11101482 (PMC6826424; doi:10.3390/cancers11101482)
Supplement: Supplementary file 1 [file cancers-11-01482-s001.zip › cancers-595132-suppl/Table S2.docx]

**Table S2. The genes for Connectivity Map (CMap) analysis.**

| **DLD-1-HMGA2 vs. DLD-1-Vector (absolute Log_2_ fold change more than 3)** | |
| --- | --- |
| **Upregulated genes** ^1^ | **Downregulated genes** |
| HMGA2, CASQ1, QPRT, S100A4, MYLK, BDNF, PRKCQ, PCOLCE2, PDE2A, LCN2 | PAH, RPL14, C3orf38, PTGS2, SEPP1, BRWD3, DSC2, PDP1, AGR2, MGST1, LARP6, CD24, ZFX, PLBD1, COL4A1, IMPACT, BRWD3, RAB8B, REG4, SCIN, NFIA, PHLDA1, CDC27, METTL7A, SH3BGRL, KIT, NPTX2, CFTR, NPNT, PBDC1, ZNF711, CBWD1, CBWD2, CBWD3, CBWD5, CBWD6, CBWD7 |
| **A549-sh-S100A4 vs. A549-sh-Control (absolute Log_2_ fold change more than 1)** | |
| **Upregulated genes** | **Downregulated genes** |
| FGA, NT5E, HNF1A-AS1, TM4SF20, HAS2, FGB, ITGA11, NAV1, P4HA3, CDA, CFH, HGD, RND1, TNS4, SYT13, SERPINE1, RNF157, ADAM19, OTTHUMG00000163706, NTRK3, KCNJ6, MIR21, EPS8, ABI3BP, ITGA5, STC1, OTTHUMG00000162005, TGM2, IFITM2, TMC7, HSPG2, CCDC146, UACA, LOC100132167, LGALS3BP, SCN1B, LXN, GRAMD1B, BDKRB1, TFPI, TANC2, BAAT, EFR3B, GLP2R, ABLIM3, PAPPA, RAB3B, APOH, BAMBI, ZFP36, FURIN, IFITM4P, WIPI1, DNAJC12, TMEM171, CAMK2N1, CP, IFITM3, CLDN2, ZC3H6, C12orf39, GREM1, LTBP1, ANPEP, C8orf4, OTTHUMG00000150105, NRP2, OTTHUMG00000132571, CCL2, PLEK2, GPX2, OTTHUMG00000013250, GPC6, SLC35D2, PLEKHA1, VLDLR, PTBP3, HAVCR1, NPY4R, OTTHUMG00000086611, SOX7, MIR4500, MSC, BDKRB2, MORC4, AMIGO2, R3HDM2, SULT2B1, DCAF7, ITGB6, C11orf86, HGSNAT, CD68, HTRA1, TIMD4, RASA2, PLSCR4, LPCAT2, BMP6, LGSN, ARRB1, PLAUR, IL13RA1, SMOC1, WBP2, PCED1B, STARD4, SMOX, ERO1L, IFNE, LTBP2, EPHB2, CDH17, CYP3A5, WISP2, LOC100506373, MYOCD | GFRA1, NR5A2, SNORD93, SDPR, MST4, VCAN, F2RL2, NDUFS2, E2F4, S100A4, SLFN11, CDCA7L, COMMD7, OTTHUMG00000152563, ANP32A, ADAM23, TSPAN7, CHML, TMED2, CDR1, UBE2R2, GPX4, PSMA4, TRIML2, SLC9A2, MIR3143, SLC16A2, DDX1, OTTHUMG00000152505, LOC643401, LOC100505817, TMEM5, FERMT1, CD38, FBN2, PRPF4, ATP5F1, PCDH9, CA8, CYFIP2, E2F5, AHR, SLC6A15, TPP1, SEH1L, OTTHUMG00000013630, C6orf141, INHBB, MYO1B, C5orf51, RAB14, FBXL21, PTPN13, BOD1, SLC25A13, SVEP1, CHORDC1 |

^1^ Due to the limitation of gene numbers (10-150 genes) for CMap query, top 10 upregulated genes were included.
